# Association between restless legs syndrome and erectile dysfunction: a systematic review and meta-analysis

**DOI:** 10.1007/s41105-026-00674-4

**Published:** 2026-07-20

**Authors:** Rafael Matos Vieira Gordilho, Nilo Jorge Carvalho Leão Barreto, Felipe Pinho E. Albuquerque Silva, João Victor Pereira Gonzalez, Nathalia Pedreira Santos de Lemos, Kenzo Ogasawara Donato, Paula Alana Morais Seixas, Gabriela Nunes E. Brito, Henrique Brasileiro Terceiro, Felipe Pereira Garrido Pazos, Rodrigo Barbosa Freire Silvão, Cristina Salles

**Affiliations:** 1 Brazilian Institute of Robotic Surgery, CME – Medical and Business Center, Av. Anita Garibaldi 1815, Suite 219, Block B, Ondina, Salvador, 40170-130 Bahia Brazil; 2 Mater Dei Hospital, Av. Vasco da Gama 6783, Engenho Velho da Federação, Salvador, 40220-005 Bahia Brazil; 3 Bahiana School of Medicine and Public Health, Av. Dom João VI 275, Brotas, Salvador, 40290-000 Bahia Brazil

**Keywords:** Restless legs syndrome, Erectile dysfunction, Sexual dysfunction, Sleep disorders, Men

## Abstract

**Objective:**

To evaluate whether patients with RLS have increased odds of ED compared with non-RLS controls.

**Methods:**

We conducted a systematic review and meta-analysis analyzing the odds of ED in patients with RLS. We included studies that: (i) evaluated adults with a diagnosis of RLS established by the pre-established criteria; (ii) included a control group without RLS; and (iii) evaluated outcomes related to the odds of ED in patients with RLS. We followed the PRISMA 2020 protocol.

**Results:**

This systematic review included a total sample of 33,740 participants. Patients with RLS had 70% higher odds of ED compared with non-RLS controls (OR = 1.70; 95% CI: 1.52–1.91; *p* < 0.001; I² = 0%). The group with severe RLS had lower odds of ED than the mild-moderate RLS group (OR = 0.67; 95% CI: 0.53–0.84; *p* = 0.0005). The RLS group presented increased odds of severe ED compared with non-RLS controls (OR = 4.51; 95% CI: 1.52–13.33; *p* = 0.006; I² = 0%), but no significant difference compared with controls for mild or moderate ED (*p* > 0.05).

**Conclusion:**

Individuals with RLS have 70% higher odds of ED compared with non-RLS controls. Severe ED was more frequent among patients with RLS, while no significant differences were observed for mild or moderate ED. These findings support ED screening in patients with RLS.

**Supplementary Information:**

The online version contains supplementary material available at https://doi.org/10.1007/s41105-026-00674-4.

## Introduction

Restless legs syndrome (RLS) is a relatively common sensorimotor neurological disorder characterized by an irresistible urge to move the legs, usually accompanied by unpleasant sensations that intensify during periods of rest, particularly at night [1–3]. Thus, RLS has a substantial impact on daily activities and quality of life in patients [1–3]. RLS is estimated to affect between 3% and 10% of the adult population, with its prevalence increasing with age, and is associated with substantial impairments in sleep quality, mental health, and overall well-being [1, 3–5]. Although the pathophysiology of RLS has not been fully elucidated, evidence indicates that dopaminergic dysfunction and alterations in iron metabolism play a fundamental role [6–8].

Erectile dysfunction (ED), defined as the persistent inability to achieve or maintain an erection sufficient for satisfactory sexual performance, also has significant repercussions on quality of life and interpersonal relationships [9]. ED is highly prevalent, particularly among older individuals, affecting more than half (52%) of men aged 40 to 70 years [9]. Recent studies suggest a potential relationship between RLS and ED, indicating increased odds and greater severity of this condition in individuals with RLS compared with controls [1, 3, 9]. The proposed mechanisms for this association include shared neurobiological alterations, such as central autonomic and dopaminergic dysfunction, as well as the indirect effects of sleep fragmentation on hormonal and vascular regulation [3, 9, 10].

Despite the accumulated evidence, current findings remain limited by small and heterogeneous samples, and there is no consensus regarding the magnitude or direction of the association between RLS and ED [3, 9]. Therefore, the present study aims to conduct a systematic review and meta-analysis to evaluate whether patients with RLS have increased odds of developing ED compared with non-RLS controls.

## Materials and methods

### Search strategy

The searches were conducted in the electronic databases MEDLINE/PubMed, Embase, The Cochrane Library, and Web of Science, combining Medical Subject Headings (MeSH), Health Sciences Descriptors (DeCS), and free-text keywords. The review was not restricted to English and also included studies published in Portuguese, Chinese, and Spanish. The conduct followed the PRISMA 2020 guidelines [11]. The search strategy used was: (“Restless Legs Syndrome” OR “restless legs syndrome” OR “Willis-Ekbom disease” OR “Ekbom syndrome” OR RLS OR “Restless Legs”) AND (“Erectile Dysfunction” OR “erectile dysfunction” OR impotence OR “sexual dysfunction” OR ED). The searches were performed on February 25, 2025. The references of eligible articles were manually examined and included when relevant.

### Inclusion and exclusion criteria

We included studies that: (i) evaluated adults with a diagnosis of RLS defined by the International Restless Legs Syndrome Study Group (IRLSSG); (ii) included a non-RLS control group; and (iii) evaluated outcomes related to the odds of ED in patients with RLS. Eligible designs included randomized clinical trials, non-randomized clinical trials, case–control studies, cohort studies, and cross-sectional studies. We excluded animal studies and conference abstracts.

### Identification and selection of studies

Two authors independently and blindly screened the titles, abstracts, and full texts of the preselected records to verify eligibility. We resolved disagreements through discussion and, when required, consultation with a third collaborator. We managed the selection process on the Rayyan platform [12].

### Data extraction and quality assessment

Two reviewers independently performed data extraction using a standardized form, and a third author verified the extracted data. The variables included author, year of publication, country of origin, study design, mean age, and BMI. We assessed the risk of bias using ROBINS-E.

### Outcomes assessed

The primary outcome was the odds of ED in patients with RLS compared with non-RLS controls.Secondary outcomes included the odds of ED in patients with mild-to-moderate RLS compared with those with severe-to-very severe RLS, as well as the odds of mild, moderate, and severe ED in patients with RLS compared with non-RLS controls. For these analyses, mild-to-moderate RLS was defined according to the IRLSSG Severity Scale or, in studies that did not use this scale, by a symptom frequency of 5–14 times per month. Severe-to-very severe RLS was defined according to the IRLSSG Severity Scale or, in studies that did not use this scale, by a symptom frequency of ≥ 15 times per month.

### Statistical analysis

The conduct followed the Cochrane Handbook for Systematic Reviews of Interventions, and reporting followed PRISMA 2020 [11, 13].

The analyses evaluated the odds of ED in patients with RLS vs. non-RLS controls. We quantified heterogeneity using I² and classified it as low (< 50%), moderate (50–74%), or high (≥ 75%). In the absence of significant statistical heterogeneity, pooled analyses were performed using a fixed-effect Mantel–Haenszel model. We performed the analyses using Review Manager (RevMan) version 5.1.7 (Cochrane Center, Denmark) and R (version 4.2.3; R Foundation for Statistical Computing, Vienna, Austria) [14, 15].

### Study registration

This systematic review and meta-analysis is registered in PROSPERO (International Prospective Register of Systematic Reviews), ID: CRD420251158461.

## Results

### Study selection and baseline characteristics

**Fig. 1:**
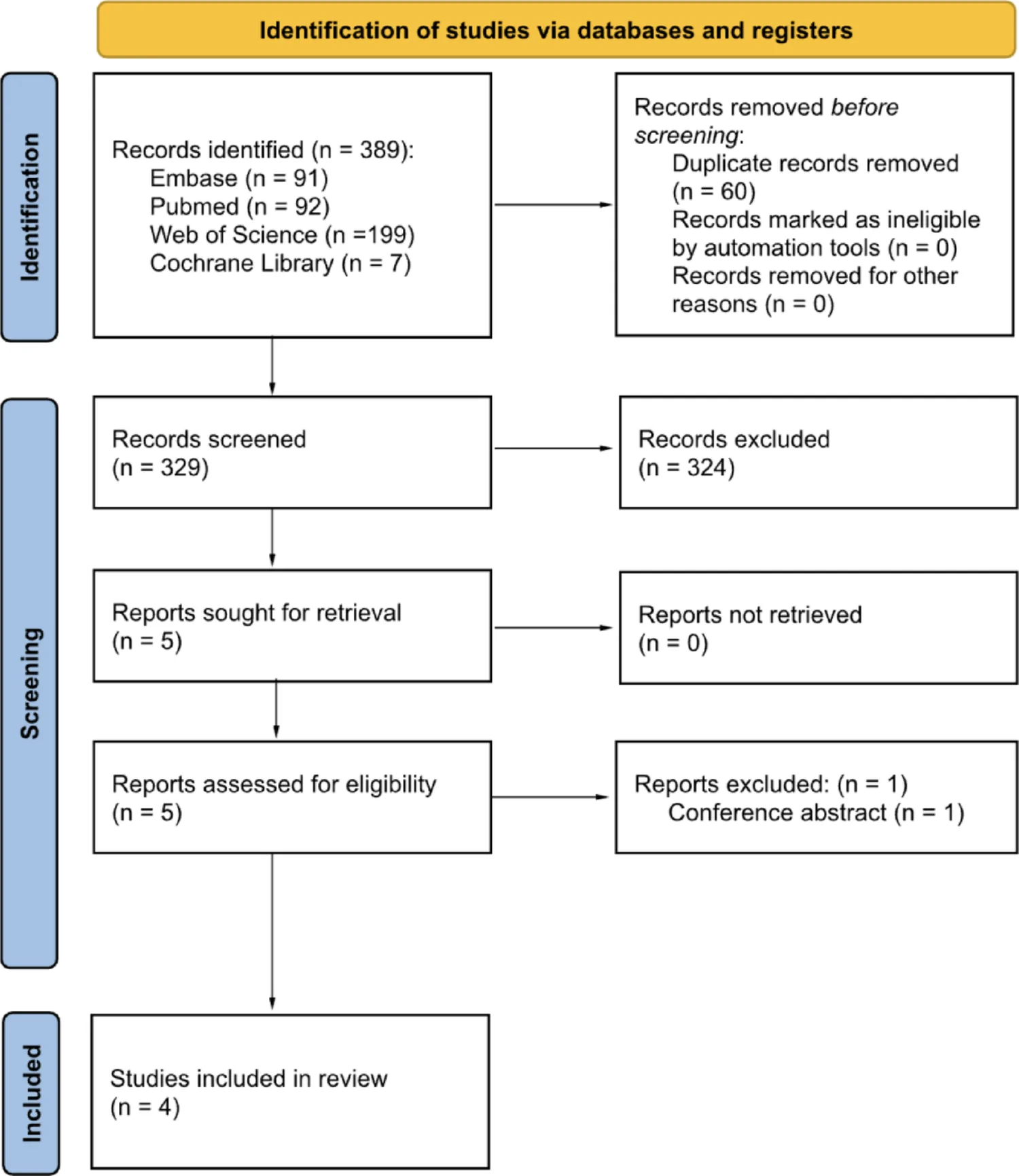
PRISMA flow diagram of study screening and selection

As detailed in Fig. 1, a total of 389 records were identified in the searched databases, and four observational studies were included in this review. The characteristics of the included studies are described in Table 1. All four articles evaluated the odds of ED in patients with RLS.

### Quality assessment

**Fig. 2:**
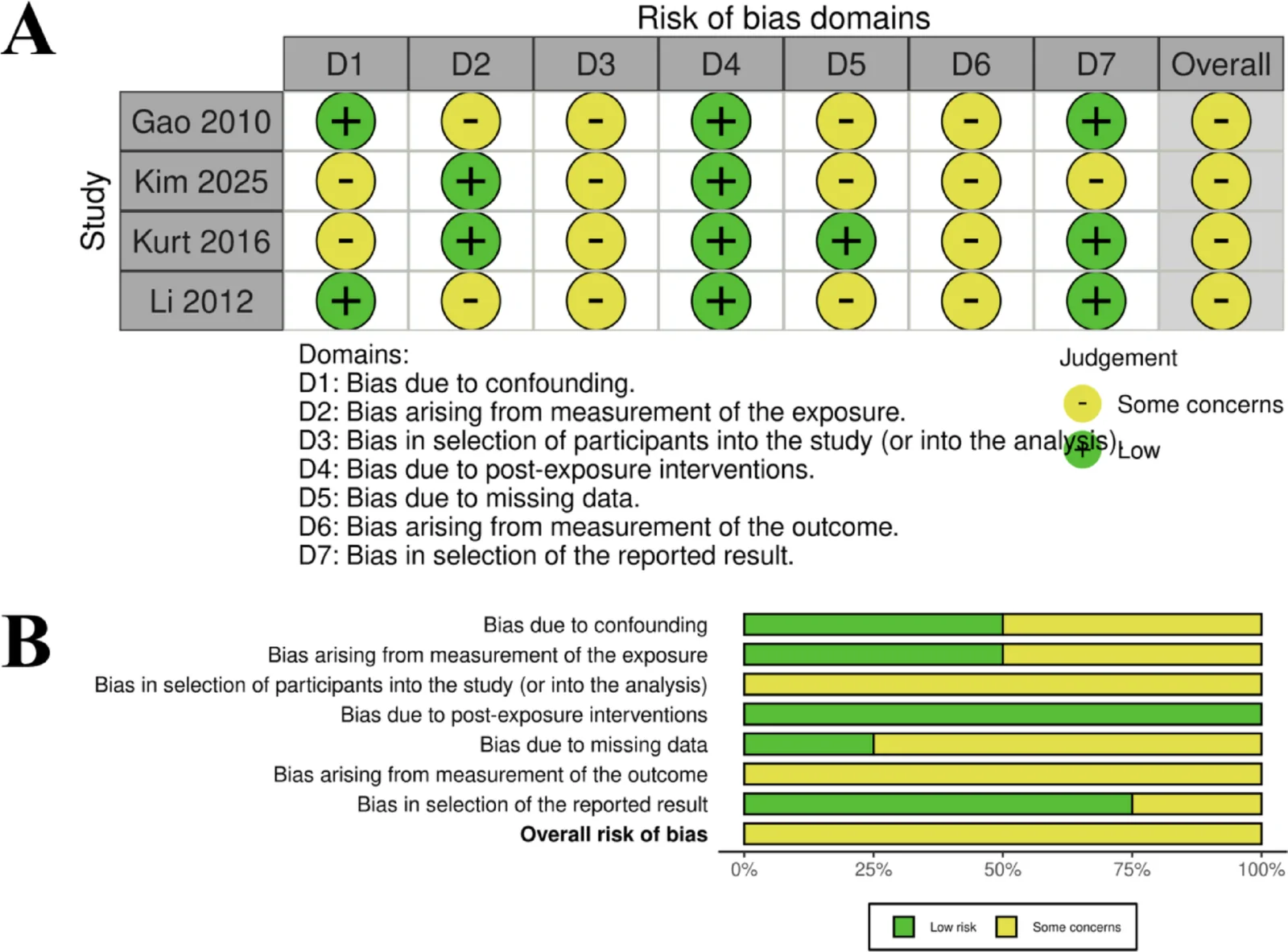
ROBINS-E of the included studies (**A**) and summary of the risk of bias (**B**)

The risk of bias in non-randomized exposure studies was assessed using the ROBINS-E tool (Fig. 2). Of the four included studies, all were considered to have “some concerns” overall. The domains that most frequently contributed to increased risk were outcome measurement (D6), selection of participants (D3), and missing data (D5). The domain-level summary (Fig. 2B) reinforces these as the main drivers of bias, with D1–D2 as secondary contributors, whereas D4 and D7 were generally at low risk.

Detailed justifications for the risk of bias assessment of each study are provided in Supplementary Table 1.

### Baseline characteristics

A total of 33,740 participants were included across four studies [1, 3, 16, 17] (Table 1), with 1372 in the RLS group and 32,368 non-RLS controls. The mean age ranged from 51.45 to 69 years, and BMI ranged from 24.38 to 27.54 kg/m².

**Table 1:** Baseline characteristics of the included studies

Author, year	Design	Country	Total *n*	Group	Mean Age (years)	BMI (kg/m²)
Gao 2010	Cross-sectional	USA	23,119	RLS = 944	69.5	26.3
				Control = 22,175	67.6	25.9
Kim 2025	Cross-sectional	Korea	127	RLS = 47	51.45 ± 14.00	25.39 ± 3.41
				Control = 80	52.34 ± 16.11	27.54 ± 4.23
Kurt 2016	Case-control	Turkey	100	RLS = 50	53.5 ± 9.9	27.4 ± 3.3
				Control = 50	53.2 ± 8.8	26.9 ± 3.4
Li 2012	Prospective cohort	USA	10,394	RLS = 331	63.4 ± 6,6	26 ± 3.26
				Control = 10,063	63.7 ± 6.9	26.4 ± 3.2

As detailed in Table 2, all included studies used the IRLSSG criteria for RLS diagnosis, although severity stratification methods varied across studies. ED assessment was performed using the International Index of Erectile Function (IIEF-5) in two studies and self-reported questionnaires in the remaining two studies. Three studies reported adjusted analyses to account for potential confounding effects.

In the present meta-analysis, clinically significant or severe RLS was harmonized according to either IRLS/IRLSSG severity scale thresholds or the presence of ≥ 15 RLS events per month, depending on the original study methodology. Adjusted effect estimates and covariates reported by the original studies were extracted to evaluate the potential impact of confounding. However, because the included studies reported heterogeneous effect measures and adjustment models, pooled analyses were conducted using crude event data extracted from each study.

**Table 2:** Methodological and diagnostic characteristics of the included studies

Author, year	RLS ascertainment	Original RLS severity definition	ED assessment	ED definition	Effect estimate	Adjusted covariates	Exclusion criteria
Gao 2010	Self-administered questionnaire (IRLSSG 2003 criteria)	Monthly frequency: 5–14 times/month vs. ≥ 15 times/month.	Self-reported questionnaire	Reports of poor or very poor ED	Adjusted OR	Age, ethnicity, smoking, weight, height, physical activity, medication use, phobic anxiety scale, and history of major chronic diseases;	Diabetes and arthritis
Kim 2025	Clinical evaluation by neurologist (IRLSSG 2014)	IRLS ≥ 15 combined with symptoms occurring > 5 nights/week = moderate-to-severe RLS	IIEF-5	Severe: 5–7 Moderate 8–11 Mild to moderate: 12–16 Mild: 17–21 No ED: 22–25	Multivariable regression	Smoking and alcohol consumption	Suspected sleep disorders by PSG; secondary RLS; medical/neurological comorbidities
Kurt 2016	Neurological assessment (IRLSSG 2003)	IRLS categories: Mild 1–10; Moderate 11–20; Severe 21–30; Very severe 31–40	IIEF-5	Severe: 5–7 Moderate: 8–11 Mild-to-moderate: 12–16 Mild: 17–21 no ED: 22–25	Crude event data	Not adjusted	No regular sexual relationship, libido loss, major psychiatric/psychological disease, alcohol/drug abuse, thyroid disease, asthma, arrhythmias, recent alpha-blockers, antipsychotics, erectile dysfunction treatment, or premature ejaculation treatment
Li 2012	Self-administered questionnaire (IRLSSG 2003 criteria)	5–14 symptoms/month; ≥15 symptoms/month	Self-reported questionnaire	Reports of poor or very poor ED	Adjusted RR	Age, ethnicity, smoking, weight and height, physical activity, medication use, phobic anxiety, lower urinary tract symptoms, history of prostate surgery, and chronic diseases	Pre-existing ED at baseline, incomplete ED data, diabetes, arthritis, prostate cancer

### Pooled analyses (all studies)

#### Odds of ED

**Fig. 3:**
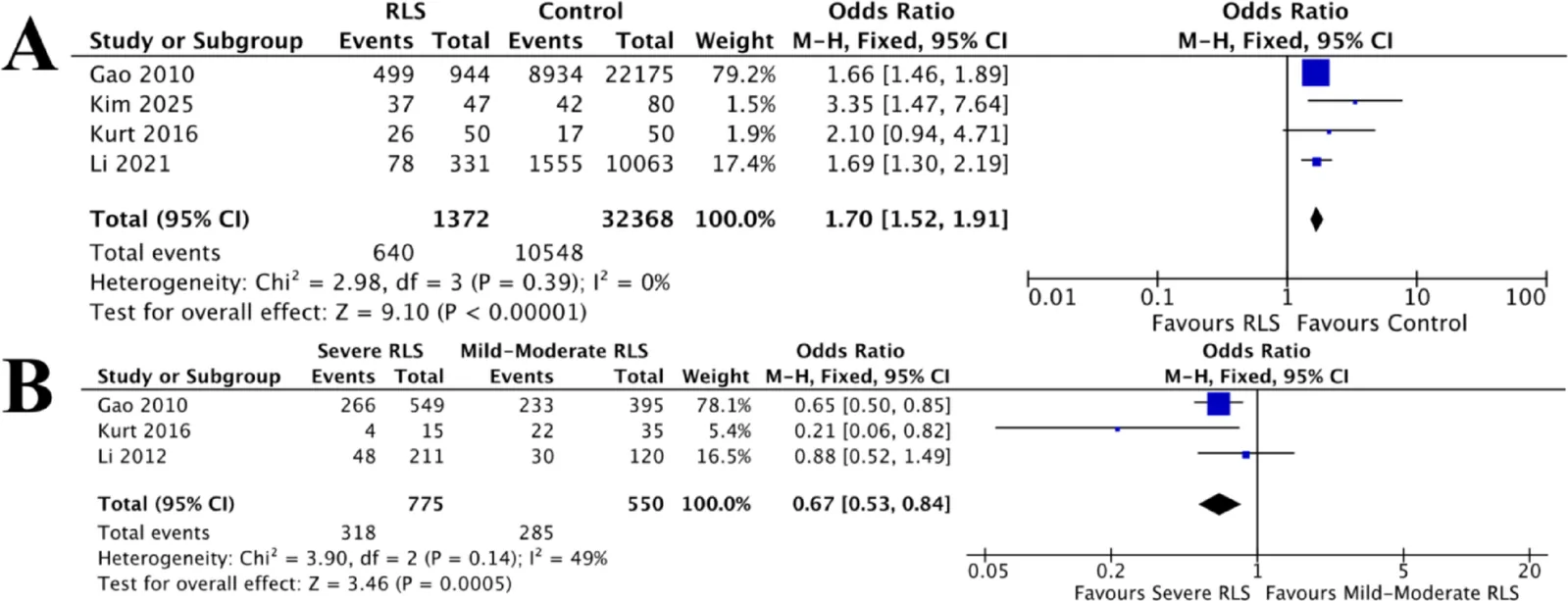
Forest plot of the odds of ED in RLS vs. non-RLS controls (**A**) and odds of ED stratified by RLS severity (**B**)

In the comparative meta-analysis of ED odds between patients with RLS and non-RLS controls, we observed higher odds of ED among individuals with RLS (OR = 1.70; 95% CI: 1.52–1.91; *p* < 0.001) (Fig. 3A), indicating that these patients had a 70% higher odds of developing ED compared with non-RLS controls, with no heterogeneity among the studies (I² = 0%).

#### Odds of ED stratified by RLS severity

The meta-analysis evaluating the odds of ED stratified by RLS severity showed lower odds in those classified as having severe RLS (OR = 0.67; 95% CI: 0.53–0.84; *p* = 0.0005) (Fig. 3B), with low heterogeneity (I² = 49%).

Given the variability in severity definitions across studies, including symptom-frequency thresholds, IRLS score categories, and combined criteria, this analysis should be considered exploratory and interpreted with caution.

#### Comparative odds of mild, moderate, and severe ED

**Fig. 4:**
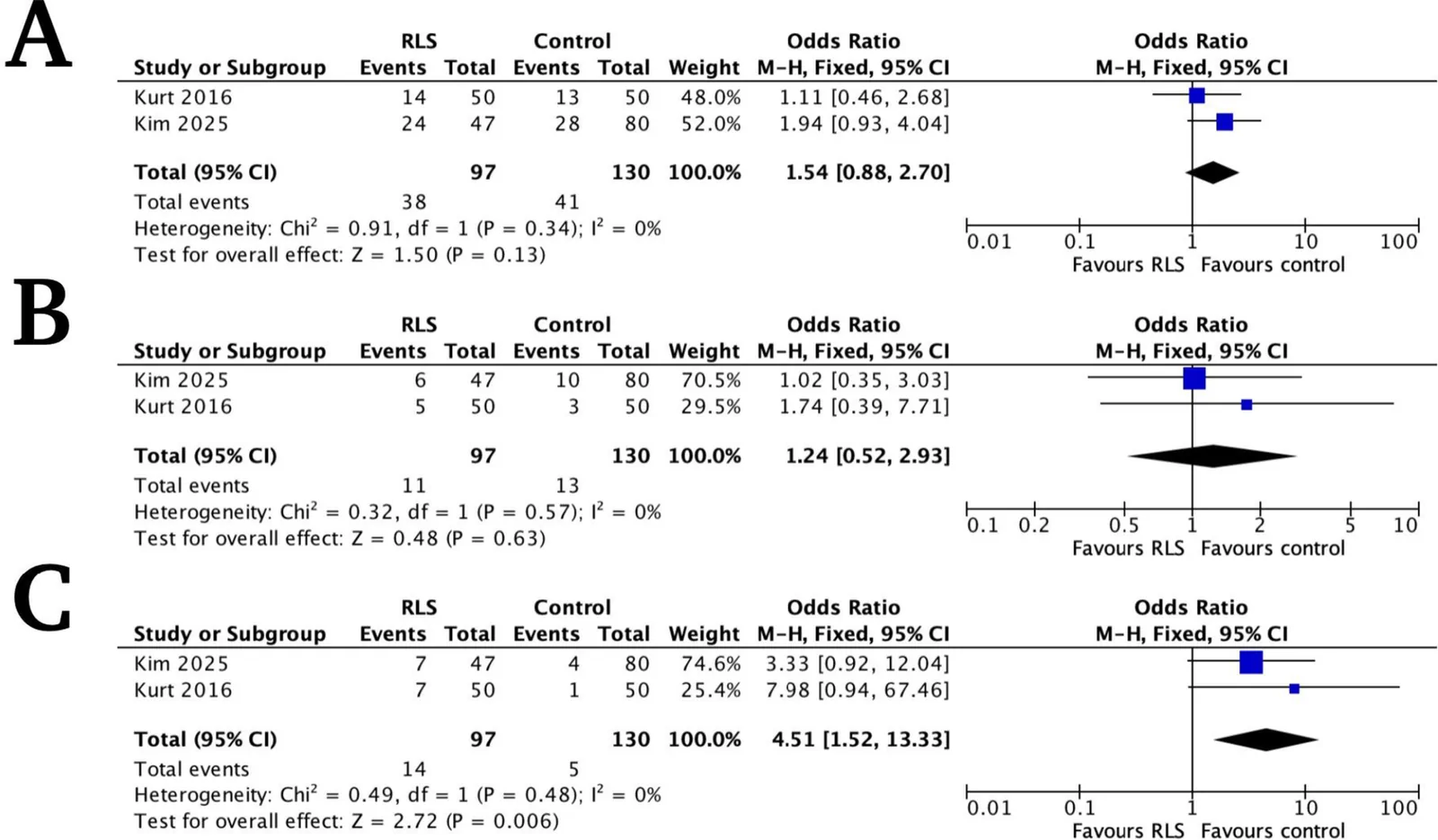
Forest plot of the odds of ED in the RLS group vs. non-RLS controls stratified by mild (**A**), moderate (**B**), and severe (**C**)

In the comparative analysis of the odds of mild ED between the RLS group and non-RLS controls, no significant difference was observed (OR = 1.54; 95% CI: 0.88–2.70; *p* = 0.13) (Fig. 4A), with no heterogeneity among studies (I² = 0%).

In the comparative analysis of the odds of moderate ED between the RLS group and non-RLS controls, no significant difference was observed (OR = 1.24; 95% CI: 0.52–2.93; *p* = 0.63) (Fig. 4B), with no heterogeneity among studies (I² = 0%).

In the comparative analysis of the odds of severe ED between the RLS group and non-RLS controls, the RLS group was associated with 351% higher odds of developing ED compared with non-RLS controls (OR = 4.51; 95% CI: 1.52–13.33; *p* < 0.001) (Fig. 4C), with no heterogeneity among studies (I² = 0%).

#### Publication bias

**Fig. 5:**
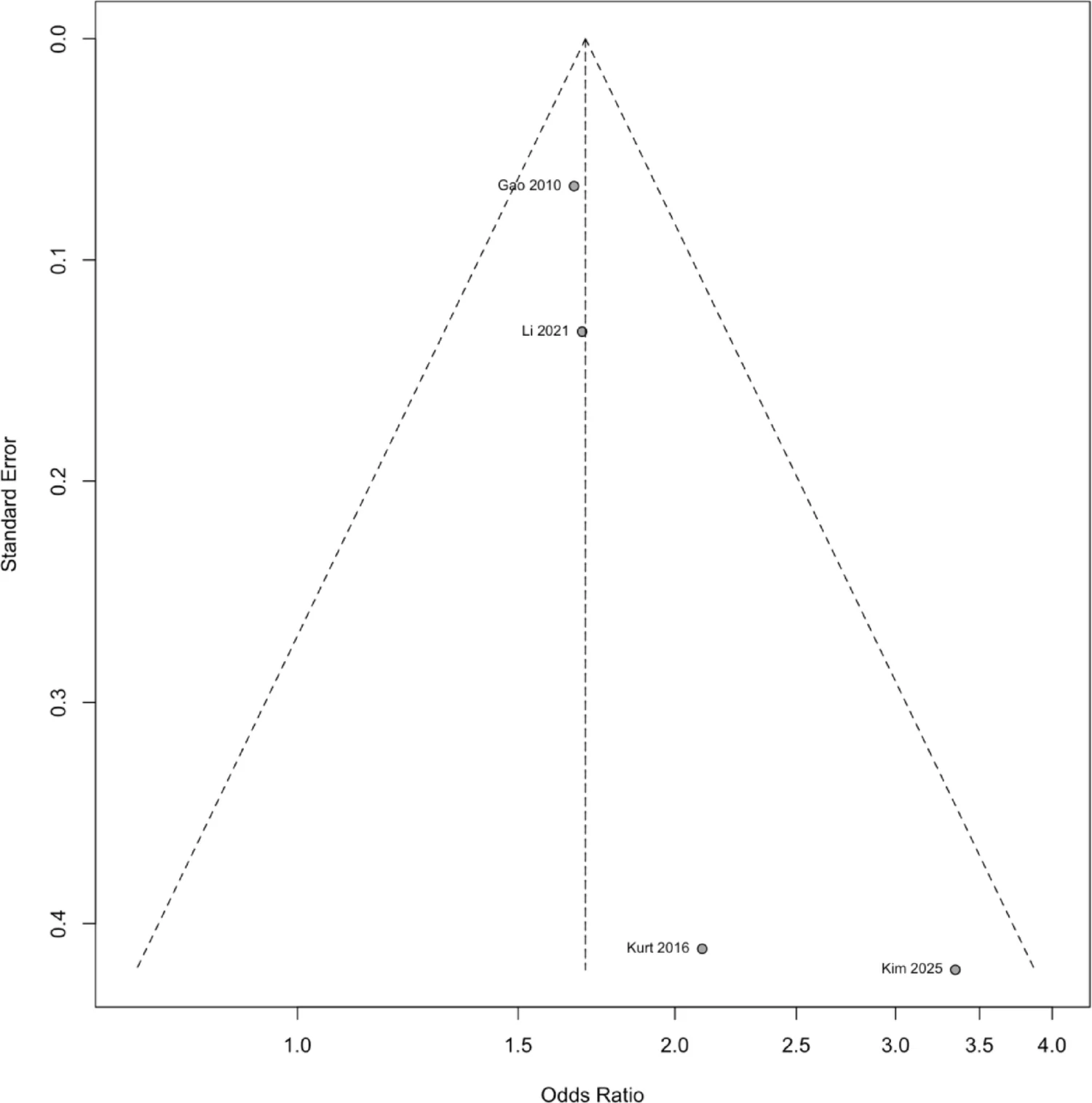
Funnel plot for the assessment of publication bias

For the assessment of the odds of ED in patients with RLS, the funnel plot demonstrated slight visual asymmetry, with smaller studies presenting larger effect estimates in favor of the RLS group. However, the limited number of included studies restricts the interpretation of this finding (Fig. 5).

## Discussion

This systematic review and meta-analysis evaluated the association between RLS and ED in a total sample of 33,740 participants. The results demonstrated that individuals with RLS had approximately 70% higher odds of ED compared with non-RLS controls. Importantly, no statistical heterogeneity was detected among studies (I² = 0%). However, this finding should be interpreted cautiously given the small number of included studies and the presence of clinical and methodological differences across study populations, diagnostic approaches, and outcome assessments.

These findings are consistent with previous observational studies, particularly those by Gao et al. [1] and Li et al. [17], which also identified a higher prevalence of ED among men with RLS. Together, these results support a possible association between RLS and ED, although the underlying mechanisms remain incompletely understood and may involve shared neurobiological, autonomic, and clinical factors.

From a pathophysiological perspective, several mechanisms may explain this association. RLS is characterized by dopaminergic dysfunction and sleep fragmentation, both of which may negatively influence sexual function. Dopamine plays a central role in the regulation of libido and penile erection, and impaired dopaminergic signaling may affect erectile pathways [18]. In addition, recurrent nocturnal arousals and increased sympathetic activity may contribute to vascular dysregulation and reduced penile blood flow [3, 19]. Because endothelial dysfunction is a key mechanism in the development of ED [20], these alterations provide a plausible biological link between both conditions.

Furthermore, the specific impact of RLS treatments and sleep comorbidities on the presence and severity of ED warrants further consideration. Given that dopamine plays a central role in the regulation of penile erection, dopaminergic therapy has a dual and paradoxical effect on male sexual function [1]. While correcting the underlying dopaminergic deficit could potentially alleviate ED symptoms, as evidenced by the pro-erectile effects of agonists like apomorphine [21], these medications also carry a significant risk of inducing impulse control disorders, including hypersexuality [22–24]. This risk complicates the clinical assessment of true erectile recovery, as increased libido or compulsive sexual behavior may mask underlying vascular or mechanical ED. Additionally, chronic sleep fragmentation acts as a critical and independent driver of ED. Reduced total sleep time profoundly suppresses testosterone levels [24, 25], whereas sleep deprivation directly damages cavernosal tissue by inducing superoxide accumulation and inhibiting nitric oxide synthase [26].

Subgroup analyses suggested that the relationship between RLS and ED may vary according to disease severity. Patients with RLS presented a higher frequency of severe ED compared with non-RLS controls. However, the severity-stratified findings should be interpreted with caution. The included studies used substantially different definitions of RLS severity, including symptom-frequency thresholds, IRLS score categories, and combined criteria, which may not represent equivalent clinical constructs across studies. Therefore, the observed association between severity categories and ED should be considered exploratory and hypothesis-generating rather than evidence of a true severity-dependent relationship.

Some limitations should be considered when interpreting these findings. First, the severity-stratified analysis should be interpreted with caution because the included studies used different definitions of RLS severity, including symptom-frequency thresholds, IRLS score categories, and combined criteria. Therefore, severity-related findings should be considered exploratory and hypothesis-generating. Second, the number of included studies was small, limiting the robustness of pooled estimates and the assessment of publication bias. In addition, several studies had cross-sectional designs, precluding causal inference regarding the association between RLS and ED.

Methodological heterogeneity was also present across studies. ED was assessed using either validated IIEF-5 instruments or self-reported questionnaires, and RLS ascertainment varied despite all studies using IRLSSG diagnostic criteria. Furthermore, pooled analyses were based on crude event data rather than adjusted effect estimates, and residual confounding from demographic, clinical, and lifestyle factors cannot be excluded. Finally, publication bias assessment was limited by the small number of included studies, reducing the reliability of funnel plot interpretation.

Despite these limitations, the available evidence suggests a potential association between RLS and ED. However, the findings should be interpreted in the context of the limited number of studies, methodological heterogeneity, and the use of crude event data in the pooled analyses. Clinicians managing patients with RLS may consider screening for ED, given the observed association between these conditions. Future prospective studies are needed to clarify the temporal relationship between RLS and ED and to investigate whether effective treatment of RLS may improve sexual function.

In summary, this systematic review and meta-analysis found that RLS was associated with higher odds of ED compared with non-RLS controls. Patients with RLS also showed higher odds of severe ED. However, severity-stratified findings should be interpreted cautiously because of differences in severity definitions across studies. Further prospective studies are needed to clarify the nature of this association and its underlying mechanisms.

## Supplementary Information

Below is the link to the electronic supplementary material.

Supplementary Material 1

## Data Availability

All authors take responsibility for all aspects of the reliability and freedom from bias of the data presented and their discussed interpretation.
