# Supplementary material for: Association between restless legs syndrome and erectile dysfunction: a systematic review and meta-analysis
Source: Sleep Biol Rhythms. 2026 Jul 20;24(4):451–9. doi: 10.1007/s41105-026-00674-4 (PMC13619993; doi:10.1007/s41105-026-00674-4)
Supplement: Supplementary file 1 [file 41105_2026_674_MOESM1_ESM.docx]

| **Supplementary Table 1. Detailed Justifications for Risk of Bias Judgments Across Domains** | |
| --- | --- |
| **Gao et al. (2010) [1]** | |
| D2 | The reliance on self-administered mailed questionnaires, without objective clinical diagnosis, precludes the rigorous exclusion of mimicking conditions (cramps/positional discomfort) and favors the potential for exposure misclassification bias |
|  |  |
| D3 | Cross-sectional design in older healthcare professionals, which precludes the establishment of temporality and causality between the variables and subjects the sample to survival bias |
|  |  |
| D5 | The absence of exposure data in 15% of the eligible sample introduces a risk of bias that was not fully mitigated by the partial analysis conducted |
|  |  |
| D6 | The outcome was assessed using a single retrospective self-report question, without the use of validated scales (such as the IIEF-5) or objective examinations; this methodological simplification increases the risk of subjectivity and misclassification error |
|  |  |
| **Kim et al. (2025) [16]** | |
| D1 | Multivariable adjustment restricted to smoking and alcohol consumption, resulting in the omission of control for fundamental variables in the association (medication use, level of physical activity, and underlying comorbidities) |
|  |  |
| D3 | Retrospective cross-sectional design conducted at a single center (tertiary hospital); the use of community advertisements for control recruitment may limit representativeness and generate selection bias |
|  |  |
| D5 | Systematic exclusion of participants without sexual activity (IIEF-5 < 5) and limitations of retrospective data for essential clinical covariates |
|  |  |
| D6 | The outcome was based exclusively on self-report (IIEF-5) in participants aware of the RLS diagnosis; the absence of blinding and validation by objective examinations favors recall bias and subjective symptom detection |
|  |  |
| D7 | Outcomes were assessed using self-administered questionnaires (IIEF-5, PEDT) and estimated timing, a methodology susceptible to subjectivity and recall bias, further exacerbated by the absence of blinding to the diagnosis |
|  |  |
| **Kurt et al. (2016) [3]** | |
| D1 | The exclusive use of univariate tests, rather than multivariable models, precludes the simultaneous control of covariates. Thus, it increases the risk of residual confounding by unadjusted clinical and lifestyle factors. |
|  |  |
| D3 | The control group consisted of a convenience sample (urological patients with asymptomatic benign pathologies); the restricted hospital-based recruitment limits representativeness and introduces potential selection bias |
|  |  |
| D6 | Outcomes were assessed using self-administered questionnaires (IIEF-5, PEDT) and estimated timing, a methodology susceptible to subjectivity and recall bias, further exacerbated by the absence of blinding to the diagnosis |
|  |  |
| **Li et al. (2012) [17]** | |
| D2 | Exposure was assessed through postal self-report without in-person clinical confirmation, a methodology vulnerable to syndrome misclassification error |
|  |  |
| D3 | The cohort consisted of older healthcare professionals (USA), resulting in a sample restriction that limits generalizability and introduces potential survival bias |
|  |  |
| D5 | Nonresponse to the RLS items in 15% of the initial eligible sample (5,692 men), combined with loss to follow-up over 6 years |
|  |  |
| D6 | Identification of incident ED cases relied exclusively on periodic self-report questionnaires, a methodology susceptible to subjectivity and outcome misclassification error |
|  |  |
